# Molecular and cellular characterization of apoptosis in flat oyster a key mechanisms at the heart of host-parasite interactions

**DOI:** 10.1038/s41598-018-29776-x

**Published:** 2018-08-21

**Authors:** Ophélie Gervais, Tristan Renault, Isabelle Arzul

**Affiliations:** 1 Ifremer, RBE-SG2M-LGPMM, Station de La Tremblade, Avenue de Mus de Loup, F-17390 La Tremblade, France; 2 Ifremer, RBE, Centre de Nantes, Rue de l’Ile d’Yeu, F-44311 Nantes, France

## Abstract

*Bonamia ostreae* has been associated with the decline of flat oyster *Ostrea edulis* populations in some European countries. This obligatory intracellular parasite persists and multiplies into hemocytes. Previous *in vitro* experiments showed that apoptosis is activated in hemocytes between 1 h and 4 h of contact with the parasite. The flat oyster uses the apoptosis pathway to defend against *B*. *ostreae*. However, the parasite might be also able to modulate this response in order to survive in its host. In order to investigate this hypothesis the apoptotic response of the host was evaluated using flow cytometry, transmission electron microscopy and by measuring the response of genes involved in the apoptotic pathway after 4 h. In parallel, the parasite response was investigated by measuring the expression of *B*. *ostreae* genes involved in different biological functions including cell cycle and cell death. Obtained results allow describing molecular apoptotic pathways in *O*. *edulis* and confirm that apoptosis is early activated in hemocytes after a contact with *B*. *ostreae*. Interestingly, at cellular and molecular levels this process appeared downregulated after 44 h of contact. Concurrently, parasite gene expression appeared reduced suggesting that the parasite could inhibit its own metabolism to escape the immune response.

## Introduction

During infection, parasites, in particular obligate intracellular parasites, are subjected to pressure from their host. They have to develop strategies to successfully install into their host without being degraded by the host defense mechanisms. Some intracellular protozoan parasites promote their installation by modulating host genes encoding proteins involved in energy metabolism, growth and signalization like *Toxoplasma gondii*^1^. Others including *Trypanosoma cruzi*, *Trypanosoma brucei* and *Giardia lamblia*, produce different antigenic variants on their surface to limit host immune response^2^. Inside parasitophorous vacuoles parasites have to face oxidative environment induced by the production of Reactive oxygen species (ROS) and nitrogen species. Some pathogens like *Perkinsus marinus* produces anti-oxidants such as peroxidase and superoxide dismutase (SOD) to escape to its destruction^3,4^.

Since 1979, the rhizarian parasite *Bonamia ostreae* has been associated with mortality events of the European flat oyster *Ostrea edulis*. Although this protozoan can be occasionally observed extracellularly in epithelium, it infects and multiplies inside the hemocytes, cells notably involved in the immunity of the bivalve. Previous *in vitro* studies have shown that *B*. *ostreae* can be internalized after 30 min of contact with hemocytes^5^. Both the hemocyte and the parasite are actively involved in the internalization^5,6^. The infection with *B*. *ostreae* is associated with a decrease of esterase activities and ROS production that could allow the parasite to survive within the hemocytes^7^. Concurrently, genes involved in the apoptotic pathway including inhibitor of apoptosis (IAP) and apoptosis inducer factor (AIF) appeared modulated suggesting that this defense mechanism is activated against *B*. *ostreae*^8–10^. Investigations carried out by flow cytometry and transmission electron microscopy (TEM) confirmed the involvement of apoptosis in the response of the flat oyster to bonamiosis^11^.

In many organisms, apoptosis is involved in several biological processes such as embryogenesis and defense against environmental stressful conditions and pathogens^12,13^. Apoptosis seems to be a highly conserved process in marine invertebrates. Various studies have indeed revealed the presence of main genes involved in the intrinsic and extrinsic pathways in the genome of the cupped oyster *Crassostrea gigas*, mussels *Mytilus galloprovincialis* and *M*. *edulis* or the manila clam, *Ruditapes philippinarum*^14–17^. For example, genes encoding various caspases were identified in manila clams and Mediterranean mussels^14,16^. Genes involved in the mitochondrial pathway including members of the Bcl-2 family have also been described in *C*. *gigas*, *R*. *philippinarum* and *M*. *galloprovincialis*^15,16,18,19^.

Partly because no complete genome is currently available for the flat oyster *O*. *edulis*, only few genes related to the apoptosis pathway have been described so far in this non-model species^8,10^. In order to improve our knowledge on this mechanism in the flat oyster, apoptotic genes were searched in transcriptomic data previously obtained. This analysis allowed us not only to describe apoptotic molecular pathways in *O*. *edulis* but also to select several candidate genes for which we developed real time PCR tools.

During the course of infection, host cells can activate apoptosis in order to limit parasite multiplication and spread into the host. Nevertheless, some parasites are able to regulate this process for their own interest. In mammals, a cycle of apoptosis inhibition and activation has been described in cells hosting protozoan parasite of the genome *Leishmania*, *Plasmodium* and *Toxoplasma*^20–23^. In contrast, few studies have investigated apoptosis modulation by parasites in marine invertebrates.

The lack of bivalve cell lines hampers the development of such studies and *in vitro* experimental conditions on freshly collected hemocytes generally do not allow exceeding 4 hours^24,25^. Hemocytes of flat oyster defend against *B*. *ostreae* through apoptosis. However, the parasite might be able to modulate this response in order to survive in its host. In order to investigate this hypothesis and to test host-parasite interactions *in vitro* after more than 4 h, we have modified available *in vitro* protocol by adding antibiotics after checking if these experimental conditions did not affect hemocyte and parasite. The apoptotic response of the host was evaluated using flow cytometry, transmission electron microscopy and by measuring the response of genes involved in the apoptotic pathway using real time PCR.

Complementary, the analysis of transcriptomic data available for the parasite *B*. *ostreae* allowed us identifying genes involved in different biological functions including cell cycle and cell death. Real Time PCR tools were developed in order to evaluate their levels of expression concurrently to flat oyster genes.

## Results

### Annotation and sequence analysis

#### Ostrea edulis

The comparison of *O*. *edulis* transcriptome sequence data with *Crassostrea gigas* protein data base allowed identifying 46 091 genes among which sequences showing more than 25% of covering were selected for gene ontology analysis.

The analysis was done under Blast2GO using GO terms related to the apoptotic pathway: “apoptotic process” (GO:0006915), “execution phase of apoptosis” (GO:0097194), “regulation of execution phase of apoptosis” (GO:1900117), “negative regulation of execution phase of apoptosis” (GO:1900118), “positive regulation of execution phase of apoptosis” (GO:1900119). After filtration, 358 contigs corresponding to 72 genes appeared related to the apoptotic pathway (Supplementary Table 1). Additionally, considering the central role of caspases in the apoptosis pathway and other genes like those in Bcl-2 family, a manual screening was also done.

**Figure 1:**
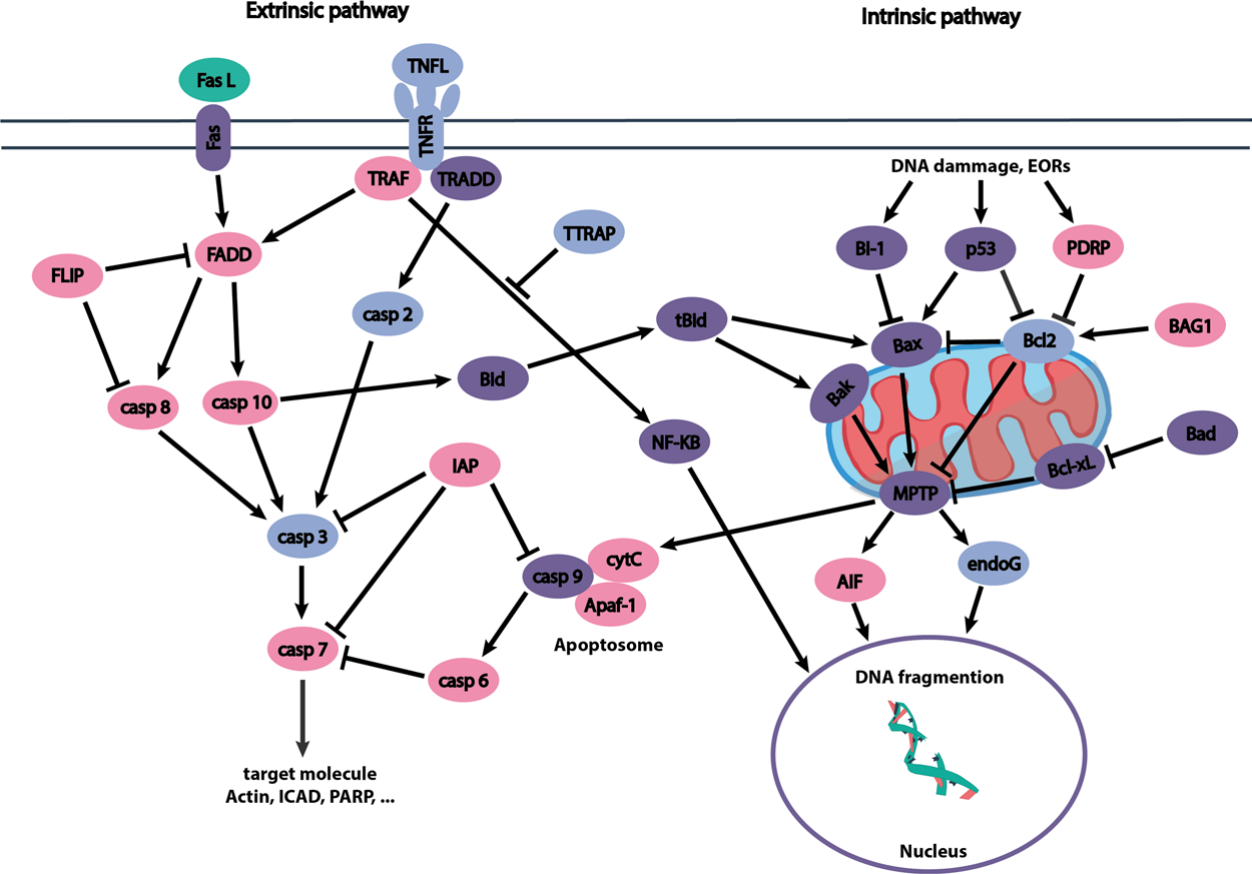
Schema representing the apoptotic pathway. Pink: genes identified in this study, light blue: genes selected for study expression measure, purple: genes not described in *Ostrea edulis*, green: gene characterised in previous study.

Gene analysis carried out in this study reveals that both intrinsic and extrinsic pathways are present in the flat oyster (Fig. 1). Caspases are central effectors of the apoptosis pathway. Except caspase 9, involved in the formation of the apoptosome, which was not identified in our data set, some genes showed maximum identity with initiator caspases (caspases 2, 8 and 10) and executioner caspases (caspases 3, 6 and 7).

A wide variety of genes involved in the extrinsic pathway were also identified including TNF - ligand and receptor, involved in the activation of the pathway and adaptors such as FADD, TRAF or TRADD.

The intrinsic pathway notably relies on the release of pro apoptotic proteins by mitochondria. Some pro-apoptotic proteins were identified in *O*. *edulis* among which members of apoptosis inducing factor (AIF) and endoG. Likewise, cytochrome C and Apaf-1, two essential elements for the apoptosome formation, required for the activation of the caspase 3, were identified in our data set.

Regulation of mitochondrial apoptotic pathway involves pro and anti-apoptotic genes belonging to the Bcl2 family. 4 homologs of Bcl2 gene were identified in *O*. *edulis* as well as two Bcl2 regulatory proteins, PDRP and BAG1.

Moreover, at least 14 different transcripts encoding inhibitors of apoptosis (IAP) were identified in *O*. *edulis* transcriptome.

#### Bonamia ostreae

Two sequences potentially related to programmed cell death were identified including (i) a gene encoding *Bax-inhibitor-1* (*BI-1*), an inhibitor of Bax protein that induces apoptosis by the release of mitochondrial content and (ii) a *Programmed cell death protein* (*PDCD*) also involved in the intrinsic pathway.

Four other putative genes were selected for their involvement in the cell cycle/cell metabolism: (i) *replication factor C* 4 (*RFC4*) involved in the elongation of primed DNA; (ii) *cell division cycle* 2 *homolog* (*CDC2H*), a catalytic subunit of the highly conserved protein kinase complex essential for G1/S and G2/M transitions; (iii) the *DNA ligase* 1 (*DNLI-1*) involved in DNA replication, recombination and the base excision repair process and (iv) Glyceraldehyde 3-phosphate dehydrogenase (GAPDH) that catalyzes the sixth step of glycolysis and thus serves to break down glucose for energy and carbon molecules.

### Evaluation of antibiotic effects on hemocytes and parasites

Prior to *in vitro* contact experiments, potential effects of antibiotics on viability and apoptosis were evaluated by flow cytometry on parasites and hemocytes, respectively. Effect on apoptosis in hemocytes was evaluated by measuring modulation of mitochondrial membrane potential and phosphatidyl serine externalization. Effect on parasite viability was estimated by measuring esterase activities and mortality. Incubation of hemocytes for 26 h with 1X and 4X of antibiotic solutions did not induce an increase of phosphatidyl serine externalization compared to the control (Table 1). Contrary to hemocytes supplemented with 1X of antibiotics, supplementation with 4X solution induced a significant increase of cells with low ∆Ψm compared to the control (Table 1).

**Table 1:** Antibiotics effects on hemocytes activities at two concentration 1X and 4X after 48 h.

	PS externalization	Low ∆Ψm
control	22.23% ± 3.97	21.04% ± 1.16
1X Ab	16.21% ± 1.15	27.78% ± 2.26
4X Ab	18.78% ± 1.02	75.33 ± 0.52 ± 0.52

Percentage of hemocytes with externalisation of phosphatidyl serine. Percentage of hemocytes with low ∆Ψm. n = 2 experiments.

While *B*. *ostreae* esterase activities were not significantly modified in presence of antibiotics, parasite mortality was reduced compared to the control (Table 2).

**Table 2:** Antibiotic effects on *Bonamia ostreae* activities and mortality.

	Esterase activities	Mortality
control	74.8% ± 6.76	18.47% ± 0.6
10X Ab	69.78% ± 2.26	7.6% ± 3.4

Percentage of parasite with esterase activities and percentage of parasite mortality. n = 2 experiments.

Based on these results, we have selected the following incubation conditions for the *in vitro* contact experiment: *B*. *ostreae* suspension was incubated in a solution of 10X of antibiotics for 15 h at 4 °C prior to be rinsed in FSW and put in contact with hemocytes. Hemocytes in contact with parasites were then supplemented with 1X of antibiotics solution.

### *In vitro* infection dynamic

**Figure 2:**
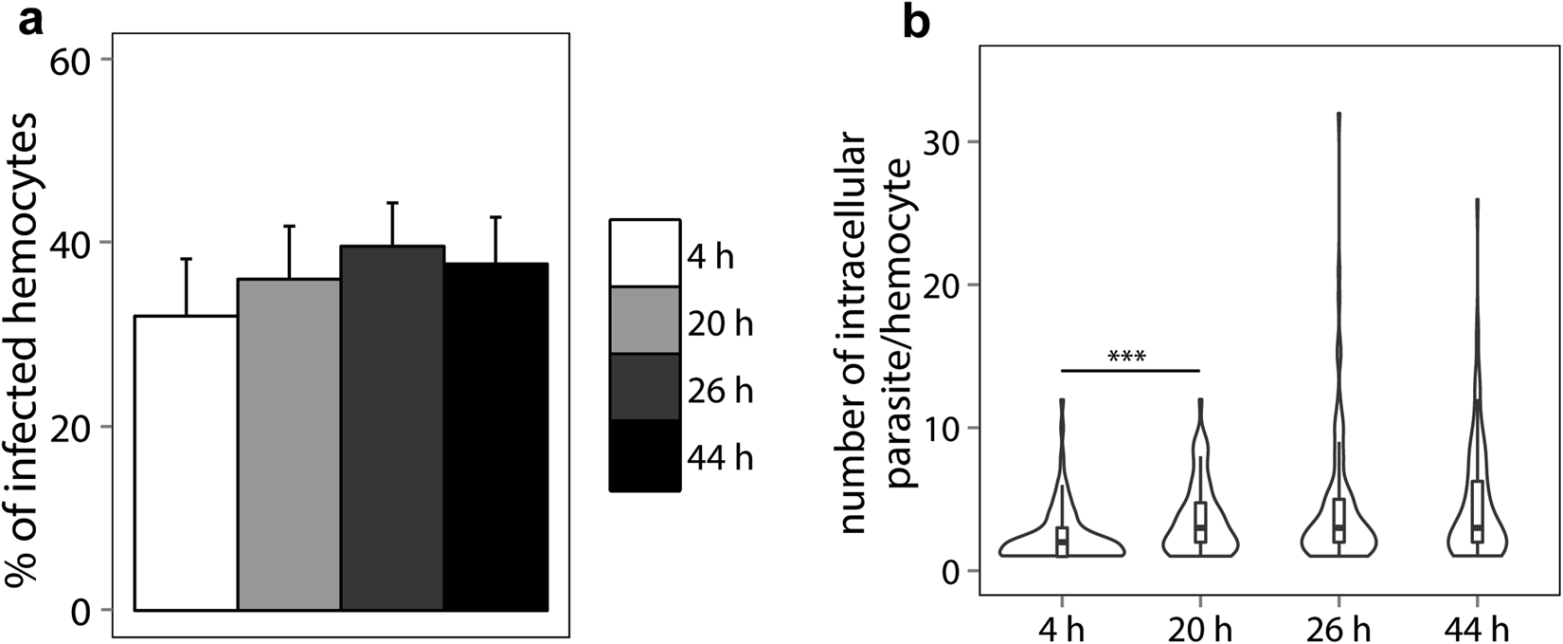
Percentage of infected hemocytes (**a**). Number of parasites per infected hemocyte (**b**). ***p < 0.001. n = 6.

Four hours after incubation with *Bonamia ostreae*, 32.1% ± 6.2 of the hemocytes have internalized at least one parasite. (Fig. 2a). This percentage remained stable between 4 (32.1% ± 6.2) and 44 h (37.8% ± 5.1). During the time of the experiment, mean number of intracellular parasites per “infected” hemocyte significantly increased from 2.4 ± 1.9 after 4 h up to 3.5 ± 2.5 after 20 h of incubation (p < 0.001) (Fig. 2b). Binucleated parasites could be observed all along the experiment.

### Effect of *Bonamia ostreae* on cellular apoptotic pathway

Involvement of apoptosis in the response of flat oyster hemocyte against the parasite *B*. *ostreae* was investigated at the cellular level by measuring phosphatidyl externalization, mitochondrial membrane potential and DNA fragmentation.

**Figure 3:**
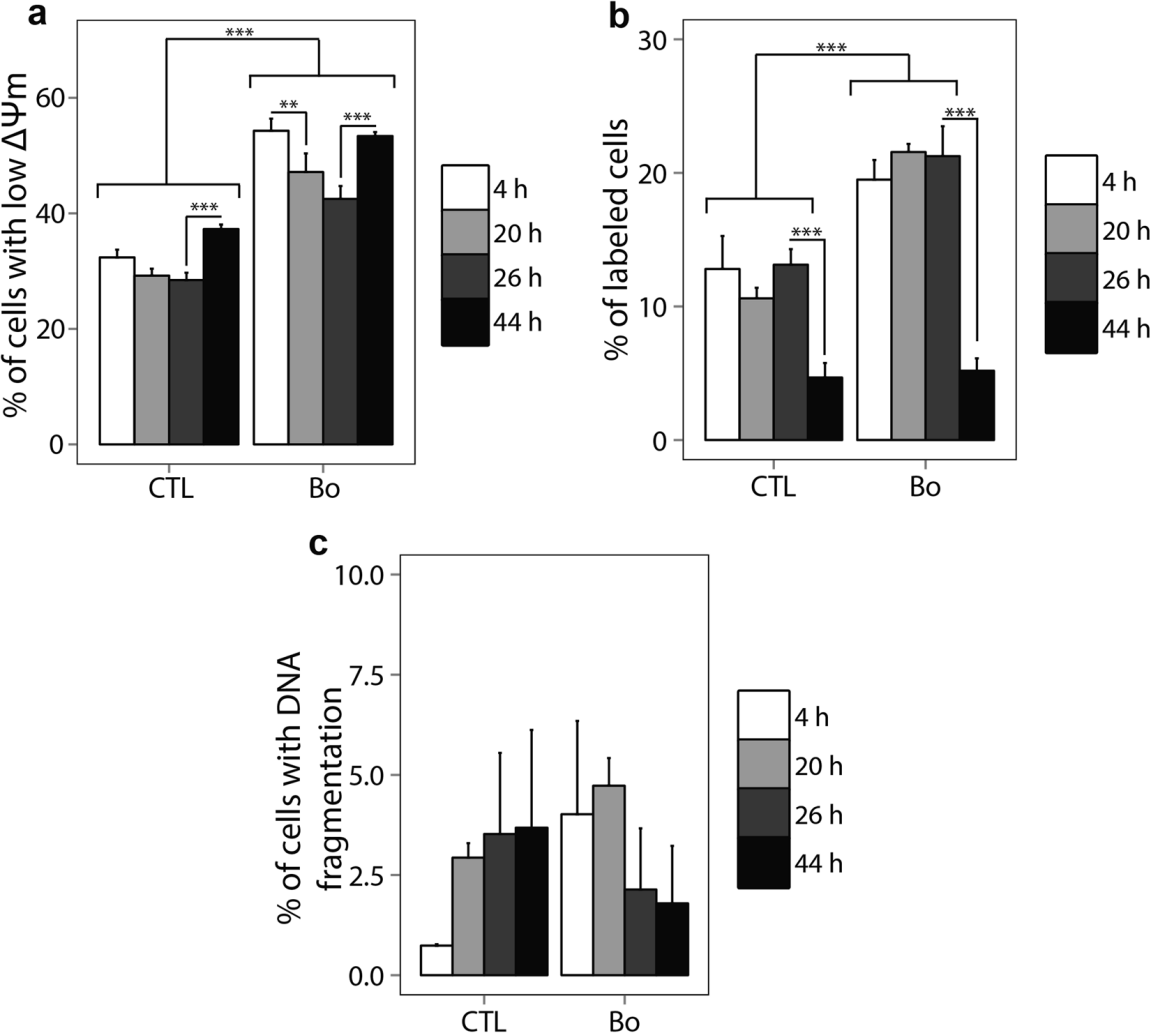
Percentage of hemocytes with low ΔΨm (**a**). Percentage of hemocytes with externalisation of phosphatidyl serine (**b**). Percentages of hemocytes showing DNA fragmentation (**c**). **p < 0.01; ***p < 0.001. n = 6.

Percentage of cells with low mitochondrial membrane potential was significantly higher in hemocytes in presence of *B*. *ostreae* compared to control hemocytes (p < 0.001) (Fig. 3a). A significant decrease (p < 0.001) of cells with low mitochondrial membrane potential was observed between 4 (54.3% ± 2.1) and 26 h (42.5% ± 2.2) of incubation with the parasite and finally it came back at 53.38% ± 0.69 at 44 h.

From 4 to 26 h the percentage of hemocytes with externalized phosphatidyl serine was significantly higher in presence of parasites compared to the control (p < 0.001) (Fig. 3b). However, it decreased at 44 h (5.19% ± 0.93) and became similar to the control (4.68% ± 1.09). The percentage of PI labeled cells did not exceed 8.3% whatever the tested conditions were.

Unlike the other tested parameters, DNA fragmentation was not significantly modulated during the time of the experiment (Fig. 3c).

### Ultrastructural changes

In order to complete results obtained in flow cytometry, hemocytes exposed to *B*. *ostreae* were also examined by TEM. Most of non-exposed cells after 4 h of incubation showed normal nucleus with non-condensed chromatin and no cytoplasmic change (Table 3). Similarly, hemocytes exposed to *B*. *ostreae* after 4 h presented few percentages of cells with ultrastructural changes associated with apoptosis. Moreover, whether the parasite was engulfed by hemocyte or not, percentage of cells showing ultrastructural changes related to apoptosis (apoptotic cells) was similar. Indeed, 22.22% of hemocytes having internalized parasite showed apoptotic features and 23.08% of hemocytes without parasite displayed ultratstructural changes. Percentages of apoptotic cells in control conditions were similar after 4 h and 20 h of incubation.

**Table 3:** Morphological changes associated with apoptosis in control (hemocytes non exposed to parasite) and hemocytes exposed to parasite (H + Bo).

		Control	H + Bo
4 h	total cells	24% (n = 50)	23,08% (n = 39)
	Cells without parasite internalization		22,22% (n = 27)
	Cells with parasite internalization		23,08% (n = 13)
20 h	total cells	30,16% (n = 63)	38,1% (n = 42)
	Cells without parasite internalization		48.39% (n = 31)
	Cells with parasite internalization		9,09% (n = 11)
44 h	total cells	64,71% (n = 51)	25% (n = 44)
	Cells without parasite internalization		35% (n = 20)
	Cells with parasite internalization		16,67% (n = 24)

Percentage of hemocytes with ultrastructural changes related to apoptosis.

In contrast, an increase of apoptotic cells was observed in *B*. *ostreae* exposed hemocytes which have not internalized parasite only. This increase of apoptotic cells was mostly observed with an increase of chromatin condensation, loss of pseudopodia and vacuolization of cytoplasm during the last stage of apoptosis. After 44 h of incubation control condition showed an increase of cells with apoptotic morphological changes (64.71%) while hemocytes exposed to *B*. *ostreae* showed a decrease of apoptotic cells. Percentage of cells with apoptotic morphological changes was still lower in hemocytes with engulfed parasites.

### Effects of *Bonamia ostreae* on *Ostrea edulis* apoptotic gene expression

Involvement of apoptosis in the response of hemocytes to *B*. *ostreae* was investigated at the molecular level by measuring the expression level of genes involved in the extrinsic and intrinsic apoptotic pathways immediately, 4, 20 and 26 h after incubation with parasites.

#### Extrinsic apoptotic pathway

**Figure 4:**
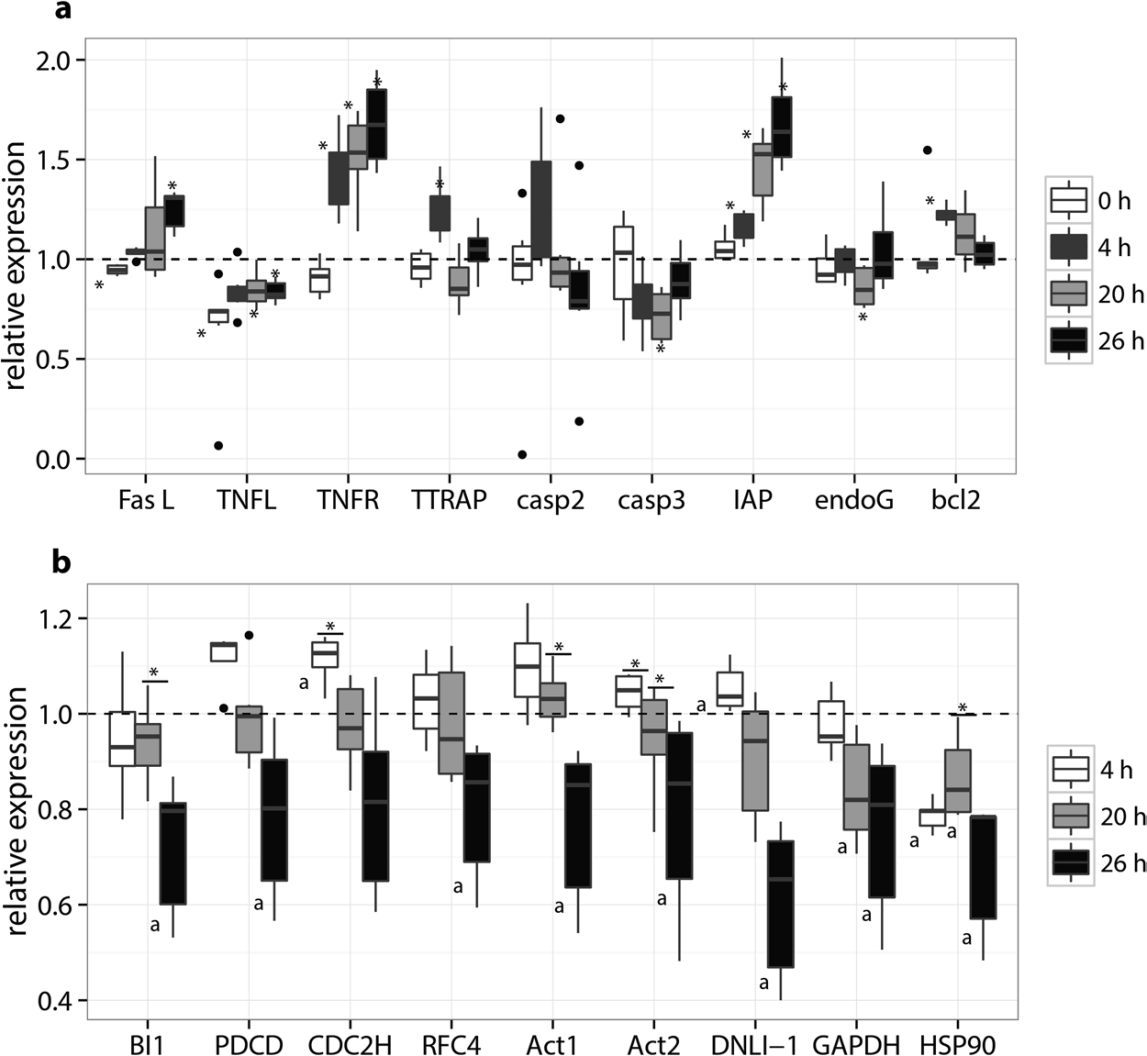
Apoptotic gene expression of *Ostreae edulis* with control hemocyte as calibrator (**a**). Gene expression of *Bonamia ostreae* with 0 h for calibrator (**b**). *p < 0.05. a: significant difference with 0 h p < 0.05. n = 6.

As soon as hemocytes were put in contact with *B*. *ostreae*, both Fas and TNFL gene expression appeared downregulated (p < 0.05) (Fig. 4a). TNFL remained downregulated all along the experiment while Fas appeared overregulated at 26 h of incubation (p < 0.05). A down regulation was followed by an overexpression (p < 0.05) of the TNF receptor and the IAP after 4 h of incubation and increased during the time of the experiment. On the contrary, expression of TTRAP was slightly modulated and showed upregulation at 4 h (p < 0.05). Genes encoding caspases did not show significant modulation except caspase 3 which appeared downregulated at 20 h of incubation (p < 0.05).

### Intrinsic apoptotic pathway

Expression of bcl2 and endoG appeared slightly modulated by the presence of parasites. Significant downregulation (p < 0.05) of endoG was observed after 20 h of contact with *B*. *ostreae* and a significant overexpression (p < 0.05) of bcl2 was noticed after 4 h of incubation (Fig. 4a).

### *Bonamia ostreae* gene expression profile

#### Housekeeping genes

Expression stability (M) of two candidate housekeeping genes was tested in our experimental conditions using two softwares: GeNorm and NormFinder. Ct values were distributed between 16.21 ± 0.27 for 18 s and 21.26 ± 0.2 for GAPDH. A low M value translates a highly stable expression in the tested conditions. Stability of these two genes was good and appeared similar using GeNorm (M = 0.03) and NormFinder (M = 0.01). Considering these results, 18 s was used as housekeeping gene to measure relative expression of other selected parasite gene.

#### Parasite gene expression

Contact of hemocytes with *B*. *ostreae* induced an early and intermittent upregulation of parasite genes including CDC2H and DNLI1 (p < 0.05) (Fig. 4b). In contrast, HSP90 appeared early and continuously down regulated in presence of hemocytes. After 26 h of contact, all genes except CDC2H were significantly downregulated (p < 0.05) compared to initial conditions.

## Discussion

Apoptosis is an highly important mechanism involved in many biological processes including defense against environmental stressful conditions or pathogens^26,27^. This mechanism allows eliminating damaged cells or cells infected with pathogens^22^. It also has a protective effect for surrounding tissues by limiting potentially damaging inflammation. For example, Pacific oyster shows an induction of apoptosis during phagocytosis of the bacteria *Planococcus citraeus*^12^.

Analysis of the flat oyster transcriptome reveals the presence of genes involved in both extrinsic and intrinsic pathways in this non-model species.

Indeed, extrinsic pathway activators including TNF receptor and ligand were identified in *O*. *edulis* as well as some adapters like FADD, TRAF or TRADD. Homologous genes were described in closely related species like FADD in Manila clam or TRAF in *Crassostrea gigas*^15,16^. Furthermore, most of initiator and executioner caspases were present in the investigated transcriptome data. These key actors have been described in various mollusc species including in *Mytilus edulis*^17^. Caspase 9, involved in apoptosome formation, was the only caspase family representative missing in our data set although capsase 9 was previously identified in *C*. *gigas* and *Ruditapes philippinarum*^15,16^.

Fewer genes involved in the intrinsic pathway were identified in the analyzed data set. Such tendency was also observed in *Mytilus edulis*^17^ whereas various genes involved in this pathway were described in other bivalve species like in *M*. *galloprovincialis* and *C*. *gigas*^15,19^. In the *O*. *edulis* transcriptome we investigated, Bcl2 and two of its regulators, PDRP and BAG1, were the only identified representatives of the Bcl2 family. Two essential components of the apoptosome, cytochrome C and the Apaf-1 were also present among filtered genes.

Interestingly, at least 14 IAPs were present in tested *O*. *edulis* transcriptome. Like in the genome of *C*. *gigas* where more than 40 IAP genes were identified, the regulation system of the apoptosis pathway seems to be an important adaptation system for the flat oyster^15,28^.

The absence of some apoptotic genes in our data set compared to other species is probably explained by the fact that the current analysis was carried out using transcriptomic sequence data and not genomic data like for *C*. *gigas*. Apoptosis is generally activated in response of environmental stressful conditions or diseases. Investigated *O*. *edulis* transcriptome was obtained from animals collected in natural beds and not exposed to particular stressful conditions. Sequencing the whole genome of *O*. *edulis* would allow improving the overview of the apoptotic mechanism in this oyster species.

Contrary to metazoans, few data are currently available on cell death process for unicellular organisms. Moreover, most of studies investigating cell death in these organisms are focused on cellular and morphological changes^29,30^. Nevertherless, few genes potentially involved in this mechanism have been described in protozoans like endonuclease G in *Leishmania major*, *L*. *infantum* and *Trypanosoma brucei*^31,32^ or programmed cell death protein in *Toxoplasma gondii*^33^. The analysis of *B*. *ostreae* transcriptome allowed us identifying two genes related to cell death and more particularly the intrinsic pathway: BI-1 and PDCD. These results suggest that a programmed cell death could exist in *B*. *ostreae*. However, additional sequence data and experimental studies are needed to investigate this mechanism in the parasite.

In mammals, apoptotic pathway can be modulated by pathogens in particular intracellular ones to survive and multiply inside the host^20–22^. Some pathogens activate the apoptosis pathway in order to decrease inflammation reaction or to kill immune cells to avoid clearance and enhance spread into its host. PS-exposing *Leishmania* promastigote after engulfment by neutrophils induces release of TGF-β that silencing their Leishmanicidal response and undergo in apoptosis^34^. On the contrary others parasites like *Plasmodium falciparum* inhibit apoptosis to avoid degradation and enhance multiplication within the target cell^35^.

In a previous study, *in vivo* experimental infection with *B*. *ostreae* was associated with a modulation of apoptotic genes including Fas ligand and IAP^8^. In addition, hemocyte apoptotic parameters appeared increased *in vitro* by the presence of parasite up to 4 h of contact^11^. These result suggest that apoptosis is involved in the defense mechanisms of the flat oyster against *B*. *ostreae*.

However this intracellular parasite infects and multiplies within hemocytes and might be able to escape degradation by inhibiting apoptosis. In order to investigate this hypothesis and host parasite interaction *in vitro* after more than 4 h we have modified a previously developed *in vitro* infection protocol by adding a mix of antibiotics. Two concentrations of antibiotics were initially tested and effects of antibiotics on hemocyte and parasites were evaluated by flow cytometry. Antibiotics adapted to kill bacteria depending of conditions can have also some effect on host cell after treatment.

Effects of antibiotics on hemocytes were evaluating through two apoptotic parameters, externalization of phosphatidyl serine and modification of mitochondrial membrane potential. No significant impact was observed when incubating hemocytes with the lower concentration of antibiotics whereas supplementation with higher concentration induced an increase of cells with low ΔΨm. Antibiotics did not modify parasite esterase activities and decreased parasite mortality. This higher parasite survival could be explained by the decrease of bacteria that can interact with *B*. *ostreae* and induce mortality.

Based on these results we subsequently incubated hemocytes and parasites with a suspension of antibiotics at the lower tested concentration. Examination of cytocentrifuged cells did not reveal bacterial proliferation in our experimental condition.

The apoptotic response of the flat oyster was followed at the cellular level using flow cytometry and microscopy tools^36^. Except DNA fragmentation, an increase of hemocyte apoptotic parameters was observed during the first hours of contact with the parasite. Like in Gervais *et al*.^11^, our results suggest that hemocytes defend against *B*. *ostreae* using the apoptotic pathway. However, the parasite seems to inhibit this phenomenon after 26 h as showed by the return of the percentage of cells with low ΔΨm to the initial value and by the decrease of phosphatidyl serine externalization. Similar results have been reported for the American oyster *C*. *virginica* infected with the parasite *P*. *marinus* which inhibits apoptosis after 24 h of contact with hemocytes^37^.

Whereas flow cytometry allow following the apoptotic process, TEM allow visualizing the consequences of this process at the ultrastructural level. At 4 h of contact no significant difference was noticed between the control and challenged hemocytes and among hemocytes exposed to the parasite, no difference was observed between “infected” hemocytes (hemocytes having at least engulfed one parasite) and none “infected” ones. More apoptotic cells were observed at 20 h of incubation both in control and challenged hemocytes. Interestingly apoptosis seems to spare “infected” hemocytes whereas it affects half of the challenged hemocytes which have not internalized parasite. After 44 h of incubation, condition appeared unsuitable for control hemocytes (in absence of parasites), 65% of them presenting apoptotic morphological changes. Increase of apoptosis in control cells was also reported for mouse spleen cells after 18 h of incubation^38^. Less ultrastructural changes were observed in presence of the parasite suggesting apoptosis inhibition mediated by the parasite itself. In other organisms it was also reported that intracellular parasites including *T*. *gondii* and *L*. *mexicana* can inhibit apoptosis of host cells even if they are treated with pro-apoptotic agents^38,39^. However, inhibition of apoptosis appeared to be more effective when the parasite was engulfed. Inhibition mechanism used by the parasite can be effective only after internalization by hemocyte and induction of apoptosis in other cells can be related to the release of cytokines by hemocytes after parasite detection. Like it was reported by TUNEL, TEM pictures of fragmented nucleus were not extensively observed. Contrary to other tested parameters, DNA fragmentation is an irreversible and the last step of the apoptosis pathway. These results suggest that the parasite can block apoptosis at the last phase.

The apoptotic response of the flat oyster to *B*. *ostreae* was also investigated at the molecular level by measuring the expression of genes involved in apoptosis, in both the extrinsic and intrinsic pathway. Upregulation of genes including Fas ligand, TNFR and TTRAP suggest that apoptosis is activated through the extrinsic pathway. However this activation is associated with an upregulation of IAP expression which probably mitigates expression of genes involved downstream in the apoptotic pathway like caspases. In our conditions, the extrinsic pathway did not seem activated as supported by the upregulation of bcl2, an inhibitor of this pathway, and the downregulation of endoG. When induced by microorganisms, apoptosis is generally induced by the recognition and binding of cytokines on receptors and follows the extrinsic pathway^12,13^. These cytokines including TNFL and FasL are produced after pathogen recognition by pattern recognition receptors (PRRs)^40^. Decrease of TNFL suggests that *B*. *ostreae* could inhibit its expression in order to block activation of the apoptosis pathway. In parallel, host cells upregulate expression of TNFR probably to compensate the decrease of TNFL. *T*. *gondii* was able to regulate expression of TNFR in order to promote the infection but this was unbalance by the overexpression of TNFL^41^. The molecular approach supports the results obtained by flow cytometry and microscopy showing an activation of apoptosis in a first step followed by an inhibition of this pathway in presence of *B*. *ostreae*. Some parasites of the genus *Leishmania* are able to block the apoptotic signal by modulating directly the endogenous signaling pathway^42^. Inhibition of apoptosis by activating expression of IAP has also been reported in *C*. *gigas* after infection by OsHV-1^43,44^.

All along the experiment the percentage of infected cells was stable and the mean number of parasites per infected hemocyte increased between 4 and 20 h. These results show that even after 44 h of contact, *B*. *ostreae* was able to survive, potentially multiplied inside hemocytes and was able to escape immune defense mechanism such as apoptosis. In order to have a better understanding of the parasite response, we have followed the expression of some *B*. *ostreae* genes involved in different biological processes including cell death and cell cycle.

An early activation of genes involved in cytoskeleton (Act1, Act2) or cell division (CDC2H) was observed supporting microscope observation: the parasite evolved and multiplied in the hemocyte. Expression of HSP90, gene notably involved in the internalization of parasites including *B*. *ostreae*^6^ appeared always below the initial expression level. Once internalized, the parasite might prevent internalisation of more parasites in order to regulate infection. Interestingly after 26 h of contact all investigated genes appeared downregulated suggesting that *B*. *ostreae* could inhibit its own metabolism to escape the immune response. This phenomenon of dormancy was reported in other parasites as a protective mechanism and can be related to a latent stage^45,46^. For example, *Plasmodium* parasite can arrest metabolic processes in order to be less sensitive to some drugs^47^.

The current study has allowed improving our general understanding of apoptosis in the non-model species *O*. *edulis*. We have not only identified genes known to be involved in both intrinsic and extrinsic pathways but also investigated their involvement in the response of oyster against the parasite *B*. *ostreae*.

Our results highlight the importance of apoptosis in the interactions between the flat oyster and the parasite *B*. *ostreae* and support a previous study in the context of which the apoptosis pathway appeared early activated as a defense mechanism against *B*. *ostreae*. However, thanks to a new adapted experimental *in vitro* protocol, we could demonstrate that after 20 h of contact, *B*. *ostreae* is able to survive and multiply within hemocytes and is able to inhibit apoptosis possibly by activating expression of IAP. Further investigations are needed to identify more precisely mechanisms developed by the parasite to control and limit apoptosis.

## Methods

### Sequence analysis and annotation

#### Ostrea edulis

Transcriptome data previously obtained from an equimolar pool of total RNA from various tissues (muscle, mantle, gills and digestive gland) of eight individual flat oysters collected from natural beds^48,49^ were used to identify apoptosis related genes. First, these sequences were compared with *Crassostrea gigas* data base using ngKLAST-LE (v4.7) and the KLASTx algorithm^50^ with threshold E-Value < 10^−3^.

In a second step, sequences showing more than 25% of covering were selected and Gene Ontology (GO) was assigned using Blast2Go^51^ software.

#### Bonamia ostreae

Transcriptome data was obtained from purified parasites *B*. *ostreae*. The cDNA libraries were prepared from 3 µg of total RNA with Illumina Tru-Seq RNA Sample Prep Kits (Illumina) for 100 bp paried-end reads according to the manufacturer’s instructions. Libraries were sequenced on a HiSeq 2000 (Illumina). The read pairs were checked and stored in ng6 environment^52^ and the transcriptome analysis was performed as described by^53^. Data were then analysed using BioMart software^54^ for gene ontology testing.

### Oysters

Flat oysters *O*. *edulis*, were collected from Quiberon bay (France) and acclimatized over 30 days in Ifremer’s facilities (La Tremblade- Charente Maritime-France). Oysters were maintained in 150 L raceway supplied with a constant flow of seawater collected from Marennes Oléron bay filtered at 1 µm and enriched with phytoplancton (*Skeletonema costatum*, *Isochrysis galbana* and *Tetraselmis suecica*).

### Hemolymph collection

Hemolymph was withdrawn from the adductor muscle sinus using 1 mL syringe. Hemolymph was kept on ice to avoid cellular aggregation and filtered at 70 µm to remove debris. Hemocyte concentration was estimated using a hemocytometer and was adjusted at 5.10^5^ cell.mL^−1^ with 0.2 µm filtered sea water (FSW).

### Parasite purification

Parasites were purified according to the protocol developed by Mialhe *et al*.^55^. Examination of gill tissue imprints by light microscopy allowed selecting oysters highly infected with *B*. *ostreae*. All the organs except the adductor muscle were homogenized and purified by differential centrifugation on sucrose gradients. *B*. *ostreae* cells were washed and resuspended in FSW and counted with a hemocytometer.

### Evaluation of antibiotics effects

Prior to *in vitro* contact experiments, the potential effects of antibiotics were evaluated on hemocytes and parasites.

Hemocytes (1 ml, 5.10^5^ cells.ml^−1^) were incubated in 24 well culture plate (Cellstar®, Greiner Bio-one) during 45 min at 15 °C. After incubation, the supernatant was removed and replaced by the same volume of FSW alone, FSW supplemented with 1X antibiotics (0,1 mg/ml of streptomycin, 0,165 mg/ml of penicillin, 0,126 mg/ml of neomycin and 0,083 mg/ml of erithromycin) or 4X antibiotics (0,4 mg/ml of streptomycin, 0,66 mg/ml of penicillin, 0,505 mg/ml of neomycin and 0,324 mg/ml of erithromycin). Modulation of mitochondrial membrane potential and phosphatidyl serine externalization on plasma membrane were monitored by flow cytometry on hemocyte suspensions after 4, 20 and 26 h at 15 °C as described below in 7.2.

Parasite suspension was incubated at 4 °C with 10 X antibiotics (1 mg/ml of streptomycin, 1,65 mg/ml of penicillin, 1,26 mg/ml of neomycin and 0,825 mg/ml of erithromycin in FSW) to avoid bacterial proliferation. After 15 h of incubation, parasites were washed in FSW. Viability and esterase activity of parasites incubated with or without antibiotics were tested by flow cytometry as described in 7.1.

### *In vitro* contact experiment

Hemocytes at 5.10^5^ cells.ml^−1^ were incubated in 24 well culture plate (Cellstar®, Greiner Bio-one) during 45 min at 15 °C. Supernatant was removed and replaced by FSW for the control or parasite suspension at a ratio of 10 parasites for 1 hemocyte. Both control and *B*. *ostreae* exposed hemocytes were supplemented with 1X antibiotics as described above. Hemocytes were incubated at 15 °C and analyzed after 4, 20, 26, 44, 48 and 70 hours. The experiment was repeated three times.

### Light microscopy

For each condition, 100 µl of hemocyte suspension were centrifuged for 1 min at 28 × g and 4 °C on slides.

Slides were stained with hemacolor® (Merk) and examined under light microscopy.

### Flow cytometry

#### Parasite survival

Effects of antibiotics on *B*. *ostreae* were tested by measuring parasite viability with propidium iodide (PI) and esterase activities with fluoresceine diacetate (FDA) by flow cytometry according to Arzul *et al*.^56^. Moreover, viability of parasite was evaluated prior to each *in vitro* contact experiment.

#### Hemocyte activity

Modulation of mitochondrial membrane potential (ΔΨm) was monitored using JC-10 dye (FluoProbes®). Externalization of phosphatidyl serine on plasma membrane and cell viability were tested using Annexin V and PI respectively (Eurobio). These activities were monitored by flow cytometry using an EPICS XL 4 (Beckman coulter) according to Gervais *et al*.^36^. Six replicates were tested for each condition.

### DNA fragmentation

For each condition, 100 µl of hemocyte suspension were centrifuged for 1 min at 28 × g and 4 °C on slides. Slides were fixed in paraformaldehyde 4% during 10 min at room temperature and kept at 20 °C after fixation. DNA fragmentation was evaluated using *In situ* Cell Death Detection Kit, POD (Roche) following Gervais *et al*.^36^.

### Transmission electron microscopy

Hemocyte suspensions were centrifuged at 1000 × g during 10 min at 4 °C and supernatant was discarded. Samples were fixed in 3% glutaraldehyde solution for 24 hours at 4 °C and processed as described in Gervais *et al*.^36^.

### Gene expression analysis

#### RNA isolation and cDNA synthesis

Hemocytes (2 mL, 5.10^5^ cells.ml^−1^) were kept in RNAlater® (Qiagen) at −80 °C until being processed. Samples were centrifuged at 4 °C during 20 min at 1500 × g and the supernatant was discarded. Total RNA was extracted using the RNAeasy®mini kit (Qiagen) following the manufacturer’s instructions. Total RNA was treated by RNase-Free DNase Set (Qiagen) to remove genomic DNA. RNA concentration was estimated using the NanoDrop 2000 (Thermo Scientific). Direct real time PCR with no retrotranscription was performed using EF primer (Supplementary Table 2) after each DNase treatment to control absence of genomic DNA. First strand cDNA synthesis was carried out from 65 ng of RNA treated using the SuperScript®III First-Strand Synthesis System (Invitrogen).

#### Selected genes

Based on *O*. *edulis* transcriptome sequence analysis, nine host genes were selected for their involvement in the apoptosis pathway. These genes were chosen to provide an overview of the apoptotic response of the flat oyster: five selected genes are involved in the extrinsic pathway (TNFL, TNFR, TTRAP, caspase 2 and caspase 3) and two are involved in the intrinsic pathway (bcl2 and endoG).

Based on *B*. *ostreae* transcriptome sequence analysis and gene sequences available from the literature^6,57^, nine parasite genes were selected to allow evaluating the behavior of the parasite inside the host cell. Selection was based on their involvement in different functions including cell cycle and programmed cell death (Table 4).

**Table 4:** General characteristics of genes used in this study.

Specie	Gene	Name	Function	Reference
*O*.*edulis*	Bcl2		Apoptosis	(Cahais *et al*.^49^)
*O*.*edulis*	endoG	Endonucléase G	Apoptosis	(Cahais *et al*.^49^)
*O*.*edulis*	TNFR	Tumor Necrosis Factor Receptor	Apoptosis	(Cahais *et al*.^49^)
*O*.*edulis*	TNFL 11	Tumor Necrosis Factor Ligand	Apoptosis	(Cahais *et al*.^49^)
*O*.*edulis*	TTRAP	TRAF and TNF receptor associated protein	Apoptosis	(Cahais *et al*.^49^)
*O*.*edulis*	Casp 2	Caspase 2	Apoptosis	(Cahais *et al*.^49^)
*O*.*edulis*	Casp 3	Caspase 3	Apoptosis	(Cahais *et al*.^49^)
*O*.*edulis*	FasL	Fas Ligand	Apoptosis	(Morga *et al*.^8^)
*O*.*edulis*	IAP	Inhibitor of Apoptosis	Apoptosis	(Morga *et al*.^8^)
*B*.*ostreae*	BI-1	Bax Inhibitor 1	Apoptosis	Arzul, Unpublished
*B*.*ostreae*	PDCD	Programme Cell Death Protein	Apoptosis	Arzul, Unpublished
*B*.*ostreae*	RFC4	Replication Factor C 4	Cell cycle	Arzul, Unpublished
*B*.*ostreae*	CDC2H	Cell Division Cycle 2 Homolog	Cell cycle	Arzul, Unpublished
*B*.*ostreae*	DNLI-1	DNA ligase 1	Ligase activity	Arzul, Unpublished
*B*.*ostreae*	HSP90	Heat Shock Protein 90	Binding protein	(Prado-Alvarez *et al*.^6^)
*B*.*ostreae*	Actin 1		Component of cytoskeletal structure	(López-Flores *et al*.^57^)
*B*.*ostreae*	Actin 2		Component of cytoskeletal structure	(López-Flores *et al*.^57^)
*B*.*ostreae*	18 s		rRNA binding	(Cochennec *et al*.^60^)
*B*.*ostreae*	GAPDH	Glyceraldehyde-3-Phosphate Dehydrogenase	NADP binding	Arzul, Unpublished

For each gene, primers were designed using Primer3 software (http://www.bioinformatics.nl/cgi-bin/primer3plus/primer3plus.cgi/) (Supplementary Table 2) and synthesized by Eurogentec.

### Real time quantitative PCR

Real time quantitative PCR was carried out in duplicate in 96-microwell plates using Mx3000 Thermocycler sequence detector (Stratagene). Amplification reactions contained 2 µL of each primer at concentrations reported in Table 2, 10 µL of Brillant III UltraFast SYBR® Green QPCR Master Mix (Agilent Technologies) and 5 µL of cDNA dilution (1/30) in a final volume of 20 µL. Amplification was carried out using standard cycling conditions: 95 °C for 3 min, followed by 40 cycles of 95 °C for 5 s and 60 °C for 10 s. Efficacy (E) was calculated for each primer pair by determining the slope of standard curve (Table 4). Efficacies were calculated according to the following equation E = 10^(1-slope)^. Expression level of selected genes was analyzed using the method described by^58^. Host gene expression was normalized using gene encoding elongation factor-1α (EF-1α) according to Morga *et al*.^59^. Calibrator consisted in control hemocytes. *B*. *ostreae* housekeeping gene was determined in our conditions by testing the most stable expression gene between two genes, 18 s^60^ and GAPDH (from the present study), using Normfinder^61^ and GeNorm softwares^62^.

### Statistical analyses

Normality of all data sets was tested using Shapiro Wilk test and homogeneity of variances were assumed using Bartlett’s and Levene test. Two-way ANOVA were used for normal data sets to determine whether significant differences existed between time and experimental treatments. Tukey’s post hoc test was used to establish where significant differences occurred within the data set. Non normal distribution data sets were analyzed using Wilcoxon test. Analyses were conducted using RStudio.

## Electronic supplementary material

Supplementary Table 1

Supplementary Table 2
