# Supplementary material for: Molecular and cellular characterization of apoptosis in flat oyster a key mechanisms at the heart of host-parasite interactions
Source: Sci Rep. 2018 Aug 21;8:12494. doi: 10.1038/s41598-018-29776-x (PMC6104086; doi:10.1038/s41598-018-29776-x)
Supplement: Supplementary file 2 — Supplementary Table 2 [file 41598_2018_29776_MOESM2_ESM.docx]

Molecular and cellular characterization of apoptosis in flat oyster a key mechanisms at the heart of host-parasite interactions

Gervais Ophélie^1^, Renault Tristan^2^, Arzul Isabelle^1*^

**^1^Ifremer, RBE-SG2M-LGPMM, Station de La Tremblade,  Avenue de Mus de Loup, F-17390 La Tremblade, France**

^2^**Ifremer, RBE, Centre de Nantes, Rue de l'Ile d'Yeu,  F-44311 Nantes, France**

***Corresponding author :** [Isabelle.arzul@ifremer.fr](mailto:Isabelle.arzul@ifremer.fr)

**Supplementary Table 2: Quantitative PCR primers used for measuring *Ostrea edulis* and *Bonamia ostreae* gene expression.**

| Genes | Sequences 5'-3' | | Primer concentration (µM) | Amplicon lenght (bp) | Tm (°C) | Efficacies (%) |
| --- | --- | --- | --- | --- | --- | --- |
| bcl2 | Forward | TCCAGACTCGGAAGAAAGGA | 4 | 263 | 84,3 | 96,3 |
|  | Reverse | GGGGAAGGATGAATGGAGAT | 4 |  |  |  |
| Casp 2 | Forward | ATGACGGTGAGGAACAGGTC | 3 | 242 | 81,2 | 98,2 |
|  | Reverse | GGATTCCGAATGGCCTTAAT | 3 |  |  |  |
| Casp 3 | Forward | GCAGTTTTTCCGGAATGAGA | 3 | 188 | 81,7 | 97,3 |
|  | Reverse | CCGATTCACTTGAGTGAGCA | 3 |  |  |  |
| TNFL 11 | Forward | TTCCTAACGTCCCGAACAAC | 4,5 | 224 | 84,3 | 97,6 |
|  | Reverse | GCCACTCAGTCATCCTCCAT | 4,5 |  |  |  |
| TNFR | Forward | ATGGCGTCCCAATTAGTCTG | 4 | 232 | 84,3 | 101,9 |
|  | Reverse | TTCGGTTCCAAATCTTCGTC | 4 |  |  |  |
| endoG | Forward | TCCATCATCAATCGAACGAA | 5 | 186 | 81,5 | 95 |
|  | Reverse | GCCGATACCCTTCAAAAACA | 5 |  |  |  |
| TTRAP | Forward | AGTTAAAGCTGCTGCACTACACC | 4 | 234 | 83,8 | 96,8 |
|  | Reverse | CTGGAATGCCTTAGATACAATCG | 4 |  |  |  |
| FasL | Forward | TTTGGGCAGTGGTGTAAGTG | 2,5 |  | 80,2 | 97,7 |
|  | Reverse | TAGCCCTGTTTCTCCACCAG | 2,5 |  |  |  |
| IAP | Forward | CTACCTCCCAGGATTGTCA | 2,5 |  | 81 | 97,3 |
|  | Reverse | CACCACTCTCCTCCATGTCA | 2,5 |  |  |  |
| EF-1α | Forward | GTCGCTCACAGAAGCTGTACC | 4 | 162 | 82,9 | 99 |
|  | Reverse | CCAGGGTGGTTCAAGATGAT | 4 |  |  |  |
| BI-1 | Forward | ACGAAAATGGCGAACAAATC | 4 | 162 | 84,6 | 98,6 |
|  | Reverse | CAGCGAGTCCCTTCTTTCAC | 4 |  |  |  |
| PDCD | Forward | GTCGAACATCGACCAATCCT | 4,5 | 284 | 80,4 | 94,8 |
|  | Reverse | CTTTTCCGCAATTTTTGCAT | 4,5 |  |  |  |
| HSP90 | Forward | GTAACGACGGCAAGCAATTT | 5 | 225 | 85,3 | 94,8 |
|  | Reverse | CGACCTCTTTCGAAGTCCAG | 5 |  |  |  |
| Actin1 | Forward | AACTTTGACCATCGGAAACG | 5 | 296 | 84,61 | 90,1 |
|  | Reverse | TAGATCCTCCGATCCAAACG | 5 |  |  |  |
| Actin2 | Forward | AGTGACGATCGGGAATGAAC | 4 | 255 | 83,1 | 105,1 |
|  | Reverse | GGAGCGATCACGTTGATTTT | 4 |  |  |  |
| RFC4 | Forward | GTCCGCTTTGGATAGGATGA | 4,5 | 195 | 81,5 | 96,1 |
|  | Reverse | CGAACAGTTCCCTTCCAAAA | 4,5 |  |  |  |
| CDC2H | Forward | GGCGAAAGAGTACGTTGAGC | 4,5 | 205 | 81 | 96,6 |
|  | Reverse | CCCAAAGTCGGCTATTTTCA | 4,5 |  |  |  |
| DNLI1 | Forward | TCGCCAAAAGATTTGATTCC | 4 | 284 | 82,2 | 90,8 |
|  | Reverse | CTGGATTTTGATCCCTCCAA | 4 |  |  |  |
| GAPDH | Forward | TCGTCTCGTAATCCGTTTCC | 3 | 172 | 88,5 | 91,4 |
|  | Reverse | GCGGCTTACCAAAACATCAT | 3 |  |  |  |
| 18s | Forward | TCAGCACTTTTCGAGAAATCAA | 4,5 | 200 | 83 | 94,6 |
|  | Reverse | CCACCATGCATAGAATCAAGAA | 4,5 |  |  |  |
